# Recent Advances in Microflow Photochemistry

**DOI:** 10.3390/molecules16097522

**Published:** 2011-09-05

**Authors:** Michael Oelgemöller, Oksana Shvydkiv

**Affiliations:** 1 School of Pharmacy and Molecular Sciences, James Cook University, Townsville, QLD 4811, Australia; 2 School of Chemical Sciences, Dublin City University, Dublin 9, Ireland

**Keywords:** photochemistry, microflow chemistry, microreactor

## Abstract

This review summarizes recent advances in microflow photochemical technologies and transformations. The portfolio of reactions comprises homogeneous and heterogeneous types, among them photoadditions, photorearrangements, photoreductions, photodecarboxylations, photooxygenations and photochlorinations. While microflow photochemistry is most commonly employed as a micro-scale synthesis tool, scale-up and technical production processes have already been developed.

## 1. Introduction

**Figure 1:**
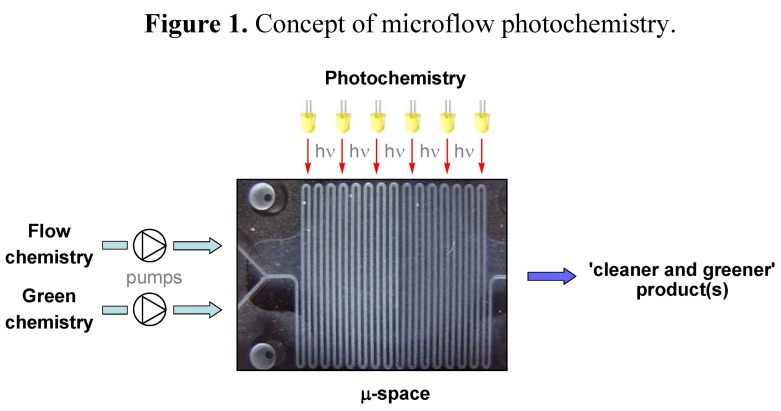
Concept of microflow photochemistry.

Synthetic organic photochemistry uses light as an energy source to initiate chemical transformations. Following this approach, a large variety of photoreactions with high selectivity, chemical yields and photon efficiencies have been developed [1,2,3]. Due to its easy generation, control and handling, light (in particular sunlight) is also considered as a ‘clean and traceless reagent’. Consequently, photochemistry has earned its place among the green and sustainable technologies [4,5,6]. Despite these advantages, however, photochemical reactions in chemical production or R&D processes are rare. Most technical processes are limited to commodity chemicals and have been developed decades ago [7,8]. Recently, miniaturized microflow devices have become widespread in synthetic chemistry [9,10,11,12,13]. The small size of the integrated reaction channel (under a millimeter in at least one dimension), together with favorable heat and mass transport, allows for precise control of reaction time, temperature, pressure and mixing. These key-features commonly result in increased selectivity, conversion and yield, and make microflow reactors advantageous for industrial [14,15] and ‘green’ processes [16,17,18,19]. ‘Microphotochemistry’ combines the advantages of microflow technology and organic photochemistry and has consequently emerged as a new synthesis concept (Figure 1) [20].

## 2. Reactor Comparison

**Figure 2:**
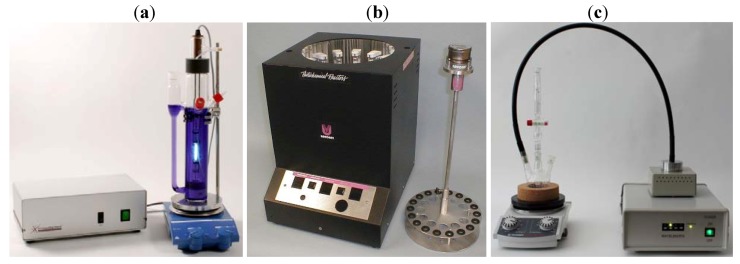
(**a**) Immersion well system (UV-LRS-1; courtesy of UV-Consulting Peschl); (**b**) Rayonet chamber reactor with merry-go-round accessory (RPR-100 with RMA-500; courtesy of Southern New England Ultraviolet Company); (**c**) Small scale reactor with light guide attachment (LUMOS 43; courtesy of Atlas Photonics).

Conventional laboratory-scale photoreactions are generally performed as batch processes using immersion well or chamber reactors (Figure 2) [7]. The total reaction volume of these laboratory systems rarely exceeds one liter. In chamber reactors, the reaction mixture is irradiated using an external array of fluorescent lamps, while immersion well systems accommodate a single low-, medium- or high pressure mercury lamp. Small scale reactor systems incorporating LEDs have also been designed and are commercially available (Figure 2c). For photoreactions in parallel, merry-go-round accessories have been developed for both reactor types.

Depending on the photophysical properties of the absorbing species, light penetration is commonly limited to a narrow layer within the reaction mixture (as expressed in the Beer-Lambert law; Equation 1). Consequently, high dilutions or narrow reaction vessels are typically applied to allow for improved light penetration. Reactions performed on micro scales can somewhat circumvent this effect due to the small size of the reaction vessels or entire reactors [21,22,23,24]:


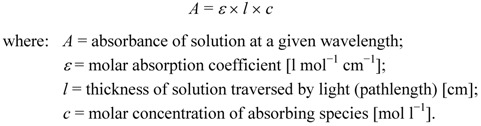
(1)

An additional limiting factor is the quantum yield at a given wavelength *Φ_λ_* (Equation 2), which describes the photon efficiency of a photochemical process and needs to be determined using actinometry [7,25]:


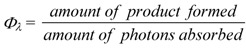
(2)

The quantum yield is unity (*Φ_λ_* = 1) if each photon absorbed by the absorbing species or chromophore yields a product molecule. Most common photoreactions have quantum yields below this optimal value due to photophysical deactivation processes (*Φ_λ_* < 1). If the quantum yield is smaller than 0.01, the conversion is very slow and exhaustive irradiation times may be required (the chemical yield of the product may still be high!). Photoinduced chain reactions can possess very large quantum yields (*Φ_λ_* >> 1) since light is only needed for the initiation step. Photochemical syntheses have a number of additional, practical limitations. Conventional lamps are rarely monochromatic and instead, show multi-wavelengths or broad (±50 nm) emissions. To avoid unwanted side or follow-up reactions, optical filters must therefore be applied. The pronounced heat release of traditional lamps furthermore requires intensive cooling procedures during operations. This, together with the limited lifetime of most lamps (~2,000 h), causes significant installation, maintenance and operation costs. Excimer lamp systems [26], lasers [27] and light emitting diodes (LEDs) [28] avoid some of these disadvantages but have other practical limitations, for example, restricted wavelengths or low power output. In addition, the batch mode with fixed volumes cannot be easily automated and follow-up reactions or decompositions due to ‘over-irradiation’ are frequently encountered. Likewise, the formation of polymeric films and iron deposits on immersion wells is commonly observed. While some smart reactor designs [29], for example falling-film [30,31], ‘liquid bell’ [32] or spinning-disc reactors [33,34], can overcome some of these drawbacks, these more sophisticated reactor types have not been widely utilized yet.

**Figure 3:**
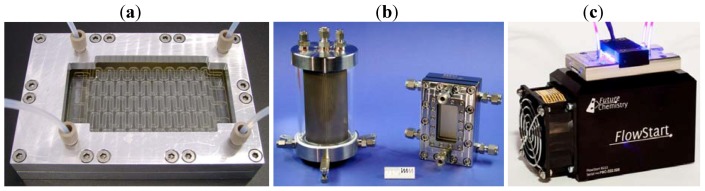
(**a**) Dwell device (courtesy of mikroglas chemtech); (**b**)Cylindrical falling-film microreactor and its standard version (FFMR-cyl and FFMR; courtesy of IMM); (**c**) Photochemistry module (courtesy of Future Chemistry).

In contrast, the design and operation features of continuous flow microreactors are particularly beneficial for photochemical transformations, especially if combined with miniaturized light-sources. Figure 3 shows a selection of commercially available microflow devices. The photochemistry module by Future Chemistry consists of a microchip and an enclosed UV-LED array on top of a cooling system. The thin layers of the reaction channels (<1,000 µm) generally ensure extensive penetration of light throughout the reaction medium even at higher concentrations of the absorbing species. The continuous flow operation furthermore prevents or reduces side reactions or decompositions caused by ‘over-irradiation’. Likewise, the irradiation period is precisely controlled via the flow rate of the pumping system. For rapid reaction monitoring, online UV- [35,36] and IR-analysis [37] have both been realized. On demand, microflow systems can be automated, up-scaled or run in parallel. Due to the efficient control of temperature, pressure and residence time, microreactors are also safer in conducting highly exothermic or potentially ‘explosive’ reactions [38].

‘Closed’ microreactors as those shown in Figure 3 have the reaction channel(s) embedded in a solid mold, for example glass, metal or plastic. The two main types are serpentine channel and falling film reactors. Serpentine channels can range from centimeters to several meters in length and may have several inlets. Some models, for example the dwell device, have additional cooling channels embedded in the reactor body (the top, parallel cooling channels are clearly visible in Figure 3a); others, for example the photochemistry module by Future Chemistry, are combined with an external cooling device (Figure 3c). Falling film reactors were specifically developed for gas-liquid reactions and contain a reaction plate with parallel microchannels [39]. The reagent gas is flowing over the liquid, falling film generated in these channels. The large interfacial area (up to 20,000 m^2^/m^3^) ensures effective saturation of the liquid film with the gas. Scale-up has been achieved using cylindrical reactor models (Figure 3b) or larger channel plates. In contrast to these ‘closed’ systems, ‘open’ reactor models use flexible microcapillaries as reaction channels [40]. Although a large range of microreactors is now commercially available, ‘in-house’ devices are still commonly constructed and utilized for photochemical studies. The reactor materials, microchannel dimensions and operation protocols can vary significantly for these ‘in-house’ systems.

## 3. Photochemical Reactions in Microreactors

Early examples of photochemical reactions under microflow conditions have been summarized independently by Oelgemöller [41] and Matsushita [42,43], respectively. This review presents an update on this exciting new methodology and features reactions reported since 2008. Wherever known, details of the batch *vs.* microreactor system are presented in tables for easy comparison. The reactions described are divided into two main categories: Homogeneous and heterogeneous reactions. While a number of (heterogeneous) catalytic applications of microflow reactors have been reported, none was used for synthesis [44,45,46,47,48,49].

### 3.1. Homogeneous Reactions

#### 3.1.1. Synthesis of Vitamin D_3_

**Scheme 1:**
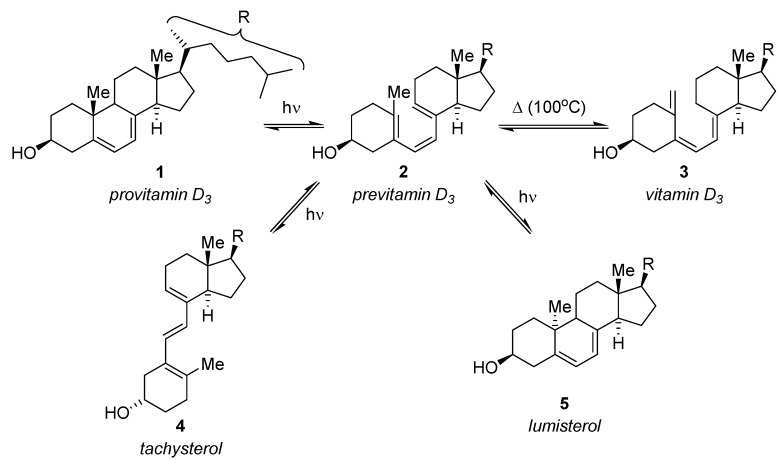
Synthesis of vitamin D_3_.

The industrially relevant synthesis of vitamin D_3_ (**3**) from provitamin D_3_ (**1**) under microflow conditions was described recently by Takahashi and coworkers [50]. The two-stage procedure involved a photochemical and a thermal reaction step (Scheme 1). The initially formed previtamin D_3_ **2** may undergo further *cis-trans* photoisomerization to tachysterol (**4**) or photoinduced electrocyclization to lumisterol (**5**).

The photochemical ring-opening of **1** to previtamin D_3_ (**2**) was initially investigated in a quartz microreactor using 1,4-dioxane, toluene and butanol as solvents, respectively. These high-boiling solvents were necessary for the subsequent thermal isomerization of **2** to vitamin D_3_ (**3**). After irradiation for 2 min or 5 min with a 400 W high-pressure mercury lamp, the crude reaction mixtures were analyzed by HPLC. Of the three solvents investigated, 1,4-dioxane gave the highest conversion (>95%) and selectivity (**2**: 45–48%; **4**: 38–39%). The formation of the undesired photocyclisation product lumisterol **5** was furthermore suppressed below 10% in this solvent. In contrast, an analogue reaction under batch conditions showed much lower selectivity (combined **2**+**4**: 23%; **5**: 25%) and conversion (recovered **1**: 50%).

The two-step synthesis was subsequently examined in two microreactors in series (Table 1) using a single 400 W high-pressure mercury lamp equipped with a Vycor filter (313–578 nm). The second reactor was additionally irradiated through a glass UV filter (360 nm) and was placed in an oil bath at 100 °C. The combined application of photochemical and thermal conditions within the second stage shifted the equilibrium of the competing photoisomerisation to tachysterol (**4**) to previtamin D_3_ (**2**) and reduced the formation of the undesired lumisterol (**5**).

A solution of provitamin D_3_ (**1**) in 1,4-dioxane (20 or 30 mM) was introduced at various flow rates with a syringe pump and the compositions of the crude reaction mixtures were determined by HPLC analysis. The highest yield of vitamin D_3_ (**3**)—60% (HPLC) or 32% (isolated)—was obtained when a 30 mM solution of **1** was used at the lowest flow rate of 5 μL min^−1^ (corresponding to an irradiation time of 10 min in the photo-microreactor and 20 min in the photo/thermal-microreactor).

The yields obtained under microflow conditions were significantly higher if compared to the industrial process, where the yield of vitamin D_3_ (**3**) is typically below 20% [7]. The continuous two-step process additionally prevented the need for purification of intermediates or high dilution conditions.

**Table 1:** Key parameters of microreactors used (no batch reactor details provided).

Photo-μ-reactor	Photo/thermal-μ-reactor
Custom-made (quartz) 1,000 μm × 200 μm × 25 cm (W × D × L) 50 μL (V_channel_)	Custom-made (quartz) 1,000 μm × 200 μm × 50 cm (W × D × L) 100 μL (V_channel_) Submerged in oil bath (100 °C)
400 W high pressure mercury lamp (Vycor filter)	400 W high pressure mercury lamp (Vycor and glass UV filter)

#### 3.1.2. Barton Reaction

**Scheme 2:**
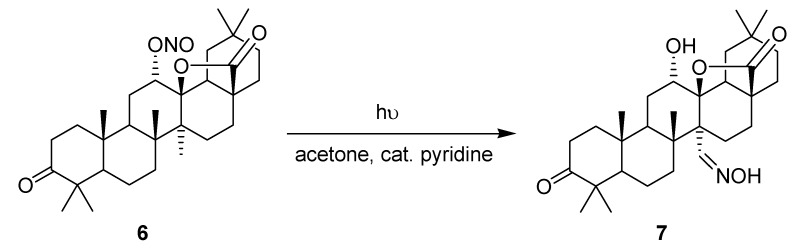
Barton reaction.

The synthesis of the steroidal oxime **7** from the nitrite precursor **6** under microflow conditions has been reported by Ryu and coworkers (Scheme 2) [51,52]. Oxime **7** is a key intermediate in the preparation of myriceric acid A, a potent endothelin receptor antagonist.

The authors initially optimized the experimental setup using a glass-covered, stainless steal microreactor (Table 2). Various glass tops were investigated using a 300 W high-pressure mercury lamp at a distance of 15 cm. An acetone solution of **6** containing catalytic amounts of pyridine was pumped through the reactor at a fixed residence time of 6 min and the crude product was analyzed by HPLC. The experiment using a soda lime cover showed the highest selectivity (19% of oxime **7**; 46% of unreacted **6**), which was explained by its favorable cut-off wavelength of >320 nm, thus avoiding photodecomposition. The optimal distance between the microreactor and the light source was subsequently examined using the soda lime top. A distance of 7.5 cm was found to be optimal and **7** was obtained in a yield of 59%. The authors additionally investigated the temperature dependence of the reaction by placing the microreactor into a temperature bath. With the soda lime cover and a distance of 15 cm between reactor and lamp, the yields of oxime **7** remained almost constant over a temperature range of 0–40 °C. Above the critical temperature of 50 °C, however, pronounced thermal decomposition of **7** was observed.

To circumvent these thermal and photochemical decomposition processes, a more compact and selective 15 W black light (UVA) lamp was chosen for further optimization studies. Subsequently, Pyrex glass and an extended residence time of 12 min gave the desired oxime product **7** in a high yield of 71% (by HPLC). Despite its lower power, the black light source gave a 10-times higher energy efficiency than the more powerful mercury lamp. A batch *vs.* microreactor comparison study was conducted in the absence of pyridine. Under microflow conditions, **7** was obtained in 56% (by HPLC) after just 6 min, whereas the batch reaction required a prolonged irradiation of 3 h to reach a similar yield of 58%. The authors furthermore examined a UV-LED panel (48 × 35 mW) as an alternative light source for the microreactor setup. Despite the low overall power output of 1.7 W, the desired product **7** was obtained in a yield of 70% (by HPLC) after a residence time of just 12 minutes.

**Table 2:** Key parameters of batch *vs.* microreactor used.

Batch	Single-lane μ-reactor
20 mL Pyrex round bottom flask 10 mL (V_solution_)	Dainippon Screen (stainless steel, glass window) 1,000 μm × 107 μm × 2.2 m (W × D × L) 0.2 mL (V_channel_)
15 W black light	15 W black light or 1.7 W UV-LED array

#### 3.1.3. [2+2]-Cycloadditions of Coumarin Derivatives

A novel flow-based photochemical reactor named LOPHTOR was recently developed by Abbott Laboratories [53]. The reactor used a pressurized fluorinated ethylene propylene (FEP) membrane to seal off the micro reaction channels. Quartz glass was utilized as a cover plate and irradiations were performed using a 450 W medium pressure mercury lamp. The reactor was connected to an automated syringe pump (auto-sampler) system that allowed for in series operation of experiments.

**Scheme 3:**
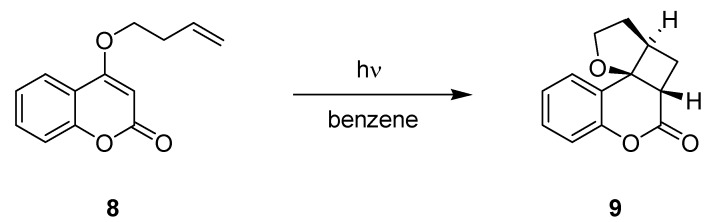
Intramolecular [2+2]-cycloaddition of coumarin derivative **8**.

The performance of the reactor was evaluated using the intramolecular [2+2]-cycloaddition of coumarin derivative **8** as a model reaction (Scheme 3; Table 3). A residence time optimization under microflow conditions was achieved using the auto-sampler unit and a 0.085 M solution of **8** in benzene. Following this approach, an optimal residence time of 2 h was established and the polycyclic product **9** was obtained in an excellent yield of 98%. A 10-fold scale-up was demonstrated for these optimized conditions with no change in the yield of **9**. In contrast, an analogue reaction performed under batch conditions in a conventional round bottom flask required 24 h of irradiation and gave product **9** in a much lower yield of 30%. The authors also launched a concentration study using a fixed residence time of 2 h. Remarkably, the conversion rates remained high with >95% over the entire concentration range of 0.085–0.425 M, thus offering a convenient access to scale-up.

**Table 3:** Key parameters of batch *vs.* microreactor used.

Batch	μ-reactor
10 mL quartz Round bottom flask 1 mL (V_solution_)	LOPHTOR (stainless steel with FEP membrane cover, quartz window) 1,000 μm × 250 μm × 3.93 m (W × D × L) 0.98 mL (V_channel_)
450 W medium pressure mercury lamp	450 W medium pressure mercury lamp (with elliptical concentrator and integral UVEXS cold mirror)

**Scheme 4:**
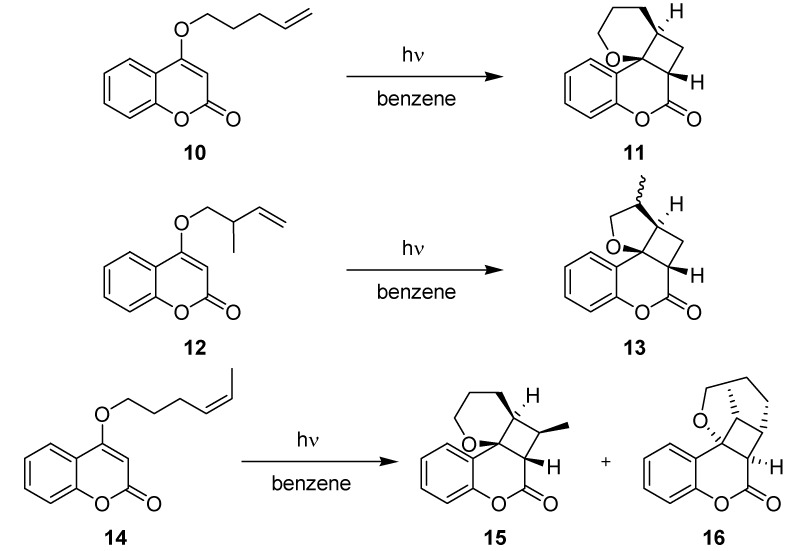
[2+2]-Cycloaddition of other coumarin derivatives.

In an extension of the work, additional coumarin derivatives were investigated under microflow and batch conditions using a fixed concentration of 0.12 M (Scheme 4; Table 4). In all cases, the LOPHTOR reactor gave higher yields and conversion rates after significantly shorter irradiation times. The diastereo- and regioselectivity were comparable in both reactor modes.

**Table 4:** Comparison of LOPHTOR *vs.* batch reaction modes.

Transformation	LOPHTOR		Batch	
	Time [h]	Yield [%]	Time [h]	Yield [%]
**10 ** **→ 11**	4	99	48	67
**12 ** **→ 13**	7	50 (1:1 ^a^)	24	30 (1:1 ^a^)
**14 ** **→ 15 + 16**	5	40 (7:1 ^b^)	24	25 (8:1 ^b^)

^a^ Diastereoselectivity; ^b^ Regioselectivity (**15** *vs.* **16**).

#### 3.1.4. Diastereoselective [2+2]-Cycloaddition of a Chiral Cyclohexenone

**Scheme 5:**
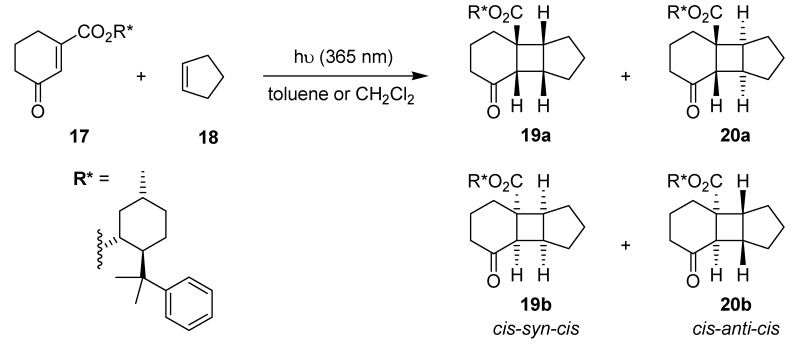
Diastereoselective [2+2] cycloaddition of cyclohexenone **17** with cyclopentene.

Kakiuchi, Ryu and coworkers have investigated the diastereoselective addition of cyclopentene **18** to the cyclohexenone derivative **17** (Scheme 5), which incorporated a chiral (−)-8-(phenyl)menthyl auxiliary [54]. Batch and microflow experiments were performed simultaneously in Pyrex vessels, which were emerged in a cooling bath, with a single 500 W high pressure mercury lamp (Table 5).

**Table 5:** Key parameters of batch *vs.* microreactor used.

Batch	μ-Reactor
Pyrex test tube 13 mm (ID) / 17 mm (OD) 2 mL (V_solution_)	Dainippon Screen (stainless steel, Pyrex window) 1,000 μm × 100 μm × 2.2 m (W × D × L) 0.2 mL (V_channel_)
500 W high pressure mercury lamp (inside a quartz immersion well)	500 W high pressure mercury lamp (inside a quartz immersion well)

Toluene and dichloromethane were tested as suitable solvents at low temperatures of 0 °C, −20 °C and −40 °C, respectively. The photoreactions in the microflow reactor generally reached completion after 30 min, whereas the batch reactions required 1 h (Table 6). The ratios of the stereoisomers **19** and **20** were determined by HPLC analysis and were almost identical for both setups in either solvent at any temperature. The preference for the formation of the *cis-anti-cis* stereoisomeric pair **20** dropped upon lowering of the temperature. At −40 °C, **19** and **20** were obtained in equal amounts for almost all reactions. The highest *d.e.* values of 82% in favor of **19a** and 54% in favor of **20a** were observed for the microflow experiment at −40 °C in toluene. In contrast, the analogue reaction performed in the test tube gave lower *d.e.* values of 72% (**19**) and 44% (**20**), respectively. The improved diastereoselectivity of the microflow synthesis was rationalized by a more accurate temperature control in combination with a significantly larger surface-to-volume ratio within the microreactor.

**Table 6:** Comparison of microreactor *vs.* batch reactions.

Reactor	Temp. [°C]	Solvent	Time [h]	Ratio19:20	*d.e.* [%]	
					19	20
μ-reactor	0	toluene	0.5	39:61	71	53
	‑20			41:59	72	53
	‑40			50:50	82	54
batch	0		1	38:62	60	37
	‑20			41:59	70	42
	‑40			50:50	72	44
μ-reactor	0	CH_2_Cl_2_	0.5	38:62	65	30
	‑20			50:50	70	32
	‑40			51:49	71	34
batch	0		1	35:65	57	27
	‑20			46:54	60	30
	‑40			50:50	67	33

#### 3.1.5. Photochemical Reduction of Flavone

**Scheme 6:**
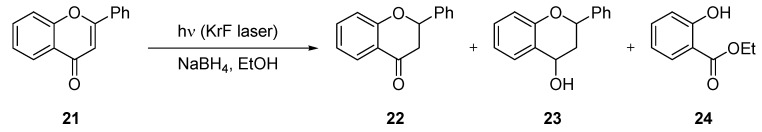
Photoreduction of flavone.

An example of a photoreduction on a non-preparative scale was reported by Ouchi and coworkers (Scheme 6; Table 7) [55]. Under batch conditions in ethanol and in the absence of light, flavone **21** remained stable in the presence of NaBH_4_. Earlier studies on the photochemistry of **21** using conventional light sources revealed the preferred formation of photodimers [56]. Upon irradiation with an excimer laser, however, photoreduction to flavanone **22**, flavanol **23** and ethyl salicylate (**24**) was observed instead. On standing after irradiation, the amount of **22** decreased due to ongoing thermal reduction to flavanol **23**. The formation of **24** increased upon prolonged irradiation indicating that it was formed by secondary photolysis from flavanone **22**. Its generation could be completely suppressed by using a single laser shot. The consumption of flavone **21** furthermore increased with increasing NaBH_4_ concentration and gave different selectivity due to competing thermal over-reduction of flavanone **22**. Consequently, no photoreduction was observed in the absence of the reducing agent. Using optimized conditions (0.5 mM of **21**; 10 mM of NaBH_4_; 1 mm optical path; 3 XeCl laser shots with 10 min intervals) the photoreduction became highly chemoselective. 93% of flavone **21** were consumed and flavanol **23** was obtained as the sole product in 57% selectivity. Reactions were additionally conducted in a masked quartz microreactor over a length of 16.5 mm. Compared to their counterparts under batch conditions, the photolyses experiments were considerable accelerated and the photoproducts **22**–**24** were formed with different selectivity.

In particular, the selectivity of **22** and **23** was slightly decreased compared to the batch reaction. These effects were explained by the differences in the optical pathlengths.

**Table 7:** Key parameters of batch *vs.* microreactor used.

Batch	μ-Reactor
Synthetic quartz cell (10 mm width and 1 mm optical path); 50 μL (V_solution_)	IMT Co. Ltd. (quartz) 100 μm × 40 μm × 16.5 mm (W × D × L ^a^)
KrF (248 nm) or XeCl (308 nm) excimer laser	KrF (248 nm) excimer laser

^a^ Microreactor masked except for a 16.5 mm × 10 mm (L × W) window.

#### 3.1.6. Photodecarboxylations Involving Phthalimides

**Scheme 7:**
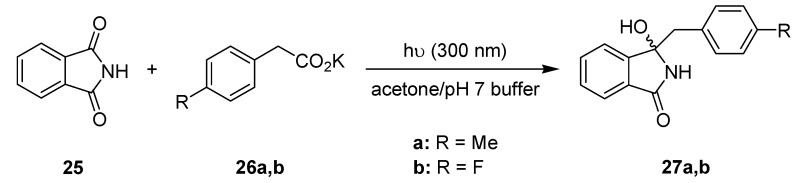
Photodecarboxylative benzylation of phthalimide.

Sensitized photodecarboxylations involving phthalimides have been established as a versatile procedure for alkylation and (macro)cyclization reactions [57,58]. Selected model reactions were also realized on semi-technical scales using an advanced falling-film reactor equipped with a 308 nm excimer lamp [30,31]. The photodecarboxylative benzylation of phthalimide **25** to the benzylated hydroxylphthalimidines **27** was subsequently investigated by Oelgemöller and coworkers under batch and microflow conditions (Scheme 7; Table 8) [59]. The sensitizer acetone functioned as a co-solvent and was thus available in large quantities (50 vol-%).

Despite its lower lamp power, the experiments conducted in the dwell device gave improved yields of 97% (**27a**) and 98% (**27b**) after a residence time of 2 h. In contrast, the batch reactions conducted in a conventional Schlenk flask furnished somewhat lower yields of 92% (**27a**) and 80% (**27b**) despite a prolonged irradiation period of 3 h. The superior performance under microflow conditions was rationalized by the better light penetration for the chromophore (and co-solvent) acetone through the narrow solution layer.

**Table 8:** Key parameters of batch *vs.* microreactor used.

Batch	μ-Reactor
Pyrex Schlenk flask (32 mm ID) with cold finger (24 mm OD) 100 mL (V_solution_)	mikroglas chemtech dwell device (Foturan™) 2,000 μm × 500 μm × 1.15 m (W × D × L) 1.68 mL (V_channel_)
Rayonet chamber reactor (RPR-200) equipped with 16 × 8 W UVB lamps	Luzchem UV panel equipped with 5 × 8 W UVB lamps

**Scheme 8:**
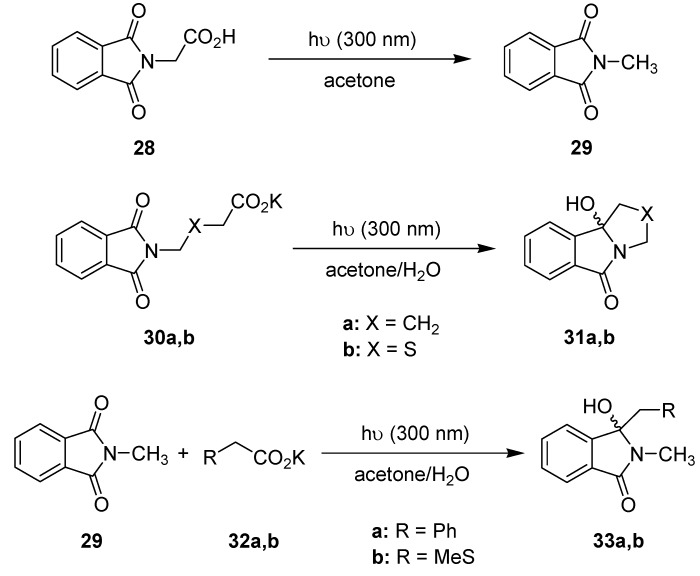
Photodecarboxylation reactions involving phthalimides.

In an extension of this work, Oelgemöller *et al.* investigated additional intra- and intermolecular photodecarboxylation reactions involving phthalimides (Scheme 8) [60]. A commercially available dwell device was again adopted as a microreactor. The volume of the batch reaction vessel varied depending on the transformation examined. In almost all cases examined, the experiments performed under microflow conditions gave superior results in terms of conversions or isolated yields (Table 9). To allow for a more precise comparison, all reactions were additionally compared using an identical light power of 40 W. Under these conditions, the microreactor setup gave between 3.5- and 6-times higher yields or conversions, respectively. The enhanced efficiency was again explained by the extensive light penetration through the narrow microchannel (500 μm). For the given acetone concentration at 300 nm, complete adsorption was achieved after approximately 1.5 mm, way below the effective pathlength of the Schlenk flask (4 mm).

**Table 9:** Comparison of dwell device *vs.* batch reaction modes.

Transformation	Time [min]	Dwell device	Batch
		Yields/Conv. [%] ^a^	Yields/Conv. [%] ^a^
**28 ** **→ 29**	3.4	5	2
	11	23	7
	21	44	19
	40	74	39
	60	92	59 (22 ^b^)
**30a ** **→ 31a**	21	33	46
	40	69	53
	60	77	69 (19 ^b^)
**30b ** **→ 31b**	21	39	36
	40	70	59
	60	80	72 (21 ^b^)
**29 + 32a ** **→ 33a**	14	83	46
	21	97	93
	40	100	100
	60	100	100 (29 ^b^)
**29 + 32b ** **→ 33b**	14	4	2
	21	34	20
	40	66	44
	60	100	86 (17 ^b^)

^a^ Conversions for reactions **28** →**29** and **29** + **32** → **33**. Yields for reactions **30** → **31**; ^b^ Batch reactor with 5 × 8 W lamps.

A concentration study was furthermore conducted using the photobenzylation of *N*-methyl phthalimide **29** as a model reaction. To achieve this, the co-solvent acetone was replaced with acetonitrile. Under these conditions, direct excitation of phthalimide **29** occurred. The reaction time was set to 1 h and the concentration of both reagents **29** and **32a** were increased systematically. The microreactor gave complete conversions at concentrations of **29** of 15 and 30 mmol L^−1^, whereas the batch system solely showed total consumption at the lowest concentration of **29** of 15 mmol L^−1^, respectively.

The same photodecarboxylation reactions were investigated using UVA light, aqueous acetonitrile solutions and 4,4’-dimethoxybenzophenone (DMBP) as sensitizer [61]. Conversion rates, isolated yields and selectivity were again compared to experiments conducted under batch conditions. In line with previous results, the dwell reactor showed superior performances. In contrast to the acetone-sensitized transformations, however, side- and follow-up reactions were additionally observed. The solid sensitizer DMBP also had to be removed by column chromatography and was sensitive towards photoreduction

#### 3.1.7. Photodecarboxylative Addition to Methyl Phenylglyoxolate

**Scheme 9:**
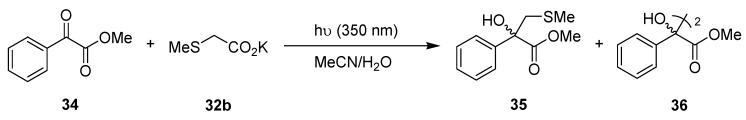
Photodecarboxylative addition to methyl phenylglyoxylate.

Oelgemöller *et al.* additionally examined the photodecarboxylative addition of sulfur containing carboxylates to alkyl phenylglyoxolates under batch conditions [62]. As part of this study, the model reaction between potassium 2-(methylthio)acetate **32b** and methyl phenylglyoxolate (**34**) was examined under microflow conditions (Scheme 9). A commercially available dwell device was again selected and compared to a larger Rayonet chamber reactor (Table 10). Using a fixed residence time of 1 h, the irradiation in microflow mode showed complete conversion and furnished the addition product **35** and the dimerization product **36** in a ratio of 76:24. When conducted in the batch reactor, an almost identical 77:23 **35**/**36** mixture was isolated. Although there was no visible difference in performance, the microreactor required significantly less lamp power and therefore showed a higher light efficiency.

**Table 10:** Key parameters of batch *vs.* microreactor used.

Batch	μ-Reactor
Pyrex Schlenk flask (32 mm ID) with cold finger (24 mm OD) 50 mL (V_solution_)	mikroglas chemtech dwell device (Foturan™) 2,000 μm × 500 μm × 1.15 m (W × D × L) 1.68 mL (V_channel_)
Rayonet chamber reactor (RPR-200) equipped with 16 × 8 W UVA lamps	Luzchem UV panel equipped with 5 × 8 W UVA lamps

#### 3.1.8. Organo-Photocatalytic Addition to Diethyl 2-Bromomalonate

**Scheme 10:**
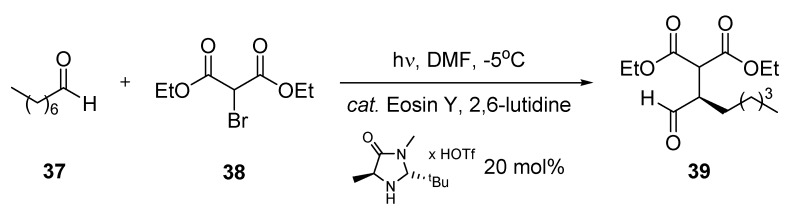
Organo photocatalytic addition to diethyl 2-bromomalonate.

An example of an ‘organic asymmetric cooperative photoredox organocatalysis’ has been briefly described by Neumann and Zeitler [63]. The stereoselective addition of octanal (**37**) to diethyl 2-bromo-malonate (**38)** was compared under microflow and batch conditions (Scheme 10; Table 11).

Eosin Y functioned as a photocatalyst and enabled the reductive dehalogenation of **38**. A chiral imidazolidinone was utilized as organocatalyst and resulted in high enantioselectivity through the formation of an enamine intermediate [64]. Irradiations were performed with green LED light at −5 °C and in the presence of lutidine. When irradiated under microflow conditions, the addition product **39** was obtained in 86% yield and with an enantiomeric excess (*e.e.*) of 87% after a residence time of 45 min. Under batch conditions, a comparable yield of 85% and a similar *e.e.* value of 88% were achieved after exhaustive irradiation for 18 h. The reduction in reaction time for the microstructured reactor was rationalized by a maximum population of excited photocatalyst within the channel.

**Table 11:** Key parameters of batch *vs.* microreactor used.

Batch	μ-reactor
Borosilicate vial (18 mm ID) 5 mL (V_solution_)	Future Chemistry photochemistry module with M-111 basic μ-reactor (Borosilicate) 600 μm × 500 μm (W_max_ × D) 100 μL (V_reaction_)
1 × 1 W 530 nm LED	2 × 1 W 530 nm LEDs

#### 3.1.9. Isopropanol Addition to Furanones

**Scheme 11:**
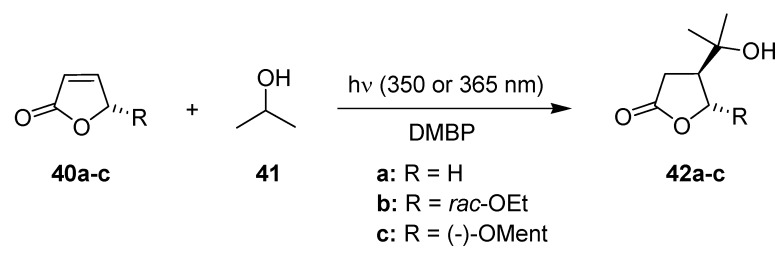
Sensitized isopropanol addition to furanones.

The DMBP sensitized addition of isopropanol (**41**) to furanones **40** has been intensively studied by Oelgemöller and coworkers (Scheme 11) [65,66,67]. The simple protocol used isopropanol as reagent and solvent, which made this transformation especially feasible for a detailed microreactor comparison study (Table 12). Three continuous-flow systems were utilized with different dimensions of the microstructure: a dwell device, a microchip and a dual-microcapillary reactor. Various residence times were investigated and the conversion rates to **42a–c** were determined by NMR-spectroscopy. The results obtained in microflow mode were compared to those obtained in a batch chamber reactor.

The batch setup, the dwell device and the microcapillary reactor gave high to quantitative conversions of furanones **40a–c** after 5 min of irradiation. Despite the much weaker power of its UV-LEDs, the microchip reached high conversions already after 2.5 min (Table 13). Reactor geometry, energy efficiency and space-time-yield (STY) calculations were additional conducted and clearly demonstrated the general superiority of all three microreactor systems over the conventional batch setup [67]. Of the three microflow designs, the LED-microchip showed the best overall performance and reaction characteristics. For example, the microchip gave the highest STY values, whereas the dwell device and the capillary reactor showed comparable STY numbers to the batch system. This finding was supported by light transmission calculations, which was most efficient for the very narrow reaction channel of the microchip (150 μm depth). Despite its improvised design, the capillary reactor was considered as the most suitable setup in terms of handling and future potential.

**Table 12:** Key parameters of batch *vs.* microreactors used.

batch	μ-Reactor	μ-Chip	μ-Capillary tower
Pyrex test tube 9 mm (ID) / 10 mm (OD) 15 mL (V_solution_)	mikroglas chemtech dwell device (Foturan™) 2,000 μm × 500 μm × 1.15 m (W × D × L) 1.68 mL (V_channel_)	Micronit Microfluidics (Borofloat^®^) 150 μm × 150 μm × 757 mm (W × D × L) 13 μL (V_channel_)	PTFE capillary (wrapped around a Pyrex cylinder) 558 μm × 460 cm (ID × L) 2 capillaries 2 × 1.12 mL (V_capillary_)
Rayonet chamber reactor (RPR-200) equipped with 16 × 8 W UVA lamps	Luzchem UV panel equipped with 5 × 8 W UVA lamps	6 × 75 mW 365 nm UV-LED array	1 × 8 W UVA lamp (inside the Pyrex cylinder)

**Table 13:** Comparison of microreactors *vs.* batch reaction modes.

	Batch		μ-Reactor		μ-Chip		μ-Capillary tower	
	Time [min]	Conv. [%]	Time [min]	Conv. [%]	Time [min]	Conv. [%]	Time [min]	Conv. [%]
**42a**	5	90	5	81	1	58	2.5	35
	20	100	10	100	2.5	89	5	75
					5	100	7.5	95
**42b**	5	90	5	99	1	60	2.5	50
	20	100	10	100	2.5	100	5	96
					5	100	7.5	99
**42c**	5	87	5	98	1	59	2.5	64
	20	100	10	100	2.5	100	5	99
					5	100	7.5	100

#### 3.1.10. Photodimerization of Maleic Anhydride

**Scheme 12:**
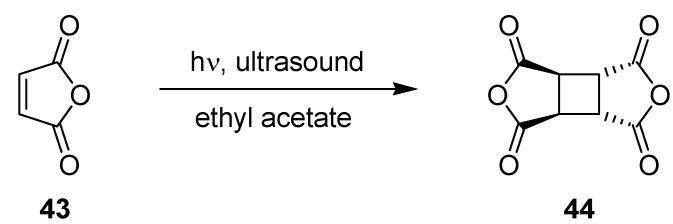
Photodimerisation of maleic anhydride.

The photodimerization of maleic anhydride (**43**) was examined by Horie *et al.* as an industrially relevant but problematic photoreaction (Scheme 12; Table 14) [68].

The photodimer cyclobutane tetracarboxylic dianhydride **44** is known to precipitate during irradiation, which can cause blockage of the reaction channel in conventional microreactors. To overcome this problem, Horie and coworkers utilized fluorinated ethylene propylene (FEP) tubes in combination with slug flow and ultrasonication. The tubing was wrapped around a tall quartz glass beaker, which was placed inside an ultrasound bath. N_2_ gas was introduced into the solution flow via a T-piece to create liquid segments of about 2–5 cm and gas segments of about 0.5–1 cm. The N_2_ segments pushed any precipitated product through the tube. Ultrasound vibrations simultaneously prevented the adhesion and sedimentation of precipitate to the tube walls. A 400 W high pressure Hg lamp in a conventional Pyrex immersion well was employed as a light source.

The impact of the tube dimensions, maleic anhydride concentration and residence time on product formation was subsequently examined. At a temperature below 15 °C and using ethyl acetate as solvent solely the *trans*-isomer of **44** was obtained, as confirmed by ^1^H-NMR analysis. A satisfactory 70% conversion of maleic anhydride (**43**) was achieved with a 10% (w/w) solution, a residence time of 22 min and an inner tube diameter of 0.8 mm, respectively. SEM analysis and subsequent polymerization experiments showed that the quality of the dimer **44** was superior over that obtained from the corresponding batch experiment. This feature was explained by the precise control of the reaction time within the microreactor, which reduced undesired side and follow-up reactions. In contrast, the batch reaction gave the desired dimer in a much lower yield of 26% after 6 h of irradiation.

**Table 14:** Key parameters of batch *vs.* microreactor used.

Batch	μ-Reactor
Cylindrical glass vessel 75 × 300 mm (ID × H) 600 g (m_solution_)	FEP tube (wrapped around a quartz beaker) 0.5–1.6 mm × 6.4–19.2 m (ID × L) 0.8–12.9 mL (V_capillary_)
400 W high pressure mercury lamp (inside a Pyrex immersion well; immersed in glass vessel)	400 W high pressure mercury lamp (inside a Pyrex immersion well; placed inside the quartz beaker)

In an interesting extension of the study, the slug flow microreactor was operated in continuous mode for over 16 h without clogging. This was achieved by inline filtration to remove the solid product **44** and reintroduction of the filtrate into the microflow reactor. A total of 300 g of a 10% solution of **43** was used for this experiment. The continuous operation mode allowed for an improvement of conversions and reduction in maleic anhydride waste.

### 3.2. Heterogeneous Reactions

Heterogeneous gas-liquid reactions require adequate supply of a reagent gas, most commonly oxygen or chlorine. This is most conveniently achieved by pre-saturation of a stock solution prior to introduction into the microreactor. Specialized reactors, for example falling film types, have furthermore been developed and successfully applied [39,69,70].

#### 3.2.1. Photooxygenation Reactions

**Scheme 13:**
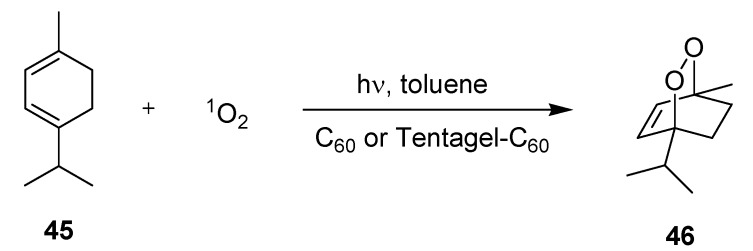
Photooxygenation of α-terpinene.

The photochemical synthesis of ascaridole (**46**) from α-terpinene (**45**) has been recently reported by Carofiglio and coworkers (Scheme 13; Table 15) [71]. The glass-polymer microstructured reactors were fabricated by the authors. C_60_ fullerene or its covalently linked form (Tentagel-C_60_) were chosen as singlet oxygen sensitizers.

The first reactor design utilized for soluble sensitizer consisted of a simple serpentine channel and two separated inlets for the substrate solution and oxygen gas, respectively. The T-mixer configuration of the gas inlet generated a microbubble flow within the channel. With the soluble sensitizer, the highest conversion of **45** of 97% (by GC) was achieved at −5 °C after a ‘correlated’ residence time of 3 min. The isolated yield of ascaridole (**46**) was, however, significantly lower with 51% and this discrepancy was explained by partial (photo)decomposition of the endo-peroxide (caused by lamp). At 10 °C and 50 °C conversions and isolated yields dropped significantly, which was rationalized by the lower solubility of oxygen in toluene at these temperatures.

The second reactor model was specifically designed for the solid-supported Tentagel-C_60_. Oxygen saturation was initially realized in a serpentine channel (so-called oxygenation zone). This solution was introduced into a wider chamber containing the solid sensitizer material. On demand, two addition chambers could be linked in series via suitable tubing. A series of reactions was subsequently conducted at −5 °C with different ‘correlated’ residence times of 27 to 109 s. Independent of the irradiation time, high conversions of **45** of >90% (by GC) and similar isolated yields of **46** of 52–53% were determined. Attempts to further reduce the residence time were unsuccessful. Likewise, irradiation with a 15 × 110 mW white LED array gave no reaction, which was attributed to its unfavorable emission spectrum in combination with the short lifetime of singlet oxygen in toluene.

Under comparable reaction conditions, the batch protocol required a significantly longer irradiation time of 1 h to reach complete conversion of **45**. The improved performance of the microreactors was explained by the favorable singlet oxygen diffusion within the narrow microchannel.

**Scheme 14:**
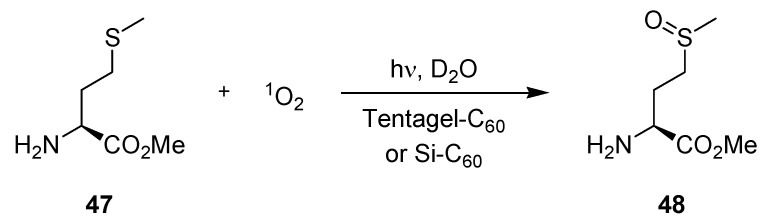
Photooxidation of L-methionine methyl ester.

The same authors investigated the S-oxidation of L-methionine methyl ester (**47**) to its corresponding sulfoxide **48** in the microflow chamber reactor (Scheme 14; Table 15) [71]. Tentagel-C_60_ and a functionalized fullerpyrrolidine-silica hybrid (Si-C_60_) were applied as sensitizing materials. The reaction was carried out in D_2_O which allowed for direct analysis by ^1^H-NMR spectroscopy. For Tentagel-C_60_ and irradiation with a conventional 300 W tungsten halogen lamp at 0 °C, conversions increased from 38–85% with extended ‘correlated’ residence time of 32–102 s. Under similar conditions with the Si-C_60_ sensitizer, complete conversions were achieved after just 33 s. When irradiated with a 15 × 110 mW white LED array at room temperature, the same silica-supported sensitizer furnished high conversions of 89% and 95% after 30 s and 42 s, respectively.

The analogue reaction under batch conditions need prolonged irradiation for approximately 1 h to achieve similar results. The improved diffusion of singlet oxygen within the microstructure was again used to explain the difference in reactivity.

**Table 15:** Key parameters of batch *vs.* microreactors used.

Batch	Serpentine μ-reactor	Chamber μ-reactor
Glass photochemical reactor 10 mL (V_solution_)	In-house (glass-thiolene) no channel dimensions given 0.152 mL (V_reactor_)	In-house (glass-thiolene) serpentine oxygenation zone500 μm (L) with chamber 3 mm × 30 mm (W × L) 0.152 mL (V_reactor_)
300 W tungsten halogen lamp	300 W tungsten halogen lamp or 15 × 110 mW white LED array	

**Scheme 15:**
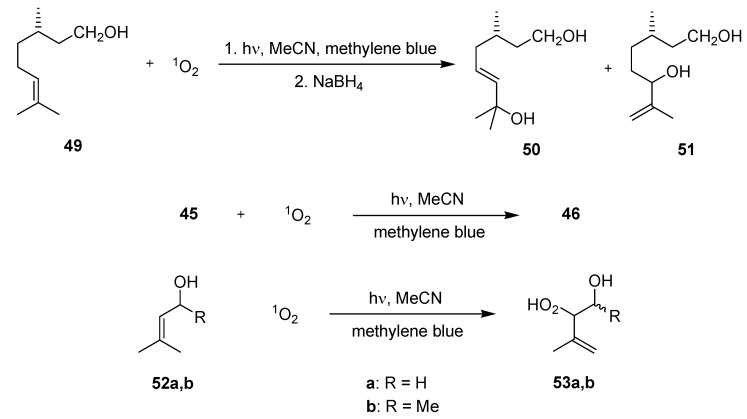
Photooxygenation reactions.

An interesting new reactor design concept was presented by Park and coworkers and tested for a range of photooxygenations (Scheme 15; Table 16) [72]. A dual-channel microreactor with a separating gas permeable membrane was constructed from poly(dimethylsiloxane) (PDMS).

The top reaction channel was coated with solvent resistant polyvinylsilazane (PVSZ) to prevent swelling of the reactor material. This ‘shielded’ material retained an optical transparency >85% between 350–700 nm. By maintaining a continuous oxygen flow, the internal pressure prevented solvent diffusion into the non-coated gas channel. For scale-up studies, a second dual-channel reactor with the liquid channel volume of 285 μL was furthermore constructed. A simple mono-channel reactor was additionally produced and was operated in plug-flow mode. Contact area-to-volume ratio calculations were subsequently conducted for the batch system, mono-channel and dual-channel reactor. The highest value of 50.9 cm^−1^ was obtained for the dual-channel, followed by the mono-channel (14.9 cm^−1^) and the batch system (0.76 cm^−1^). Three model photooxygenations were subsequently investigated in acetonitrile at 5 °C using methylene blue as sensitizer and a 16 W LED spot as light source.

**Table 16:** Key parameters of batch *vs.* microreactor used.

Batch	Mono-channel μ-reactor	Dual-channel μ-reactor
50 ml round bottom flask 4.4 cm (OD_max_) 20 mL (V_solution_)	In-house (PDMS, PVSZ coating in reaction channel) concave reaction channel 220 μm × 0.9 m (W × L) 38.9 μL (V_channel_) segment flow	In-house (PDMS with PDMS membrane, PVSZ coating in reaction channel) concave reaction channel 220 μm × 0.9 m (W × L) 38.9 μL (V_channel_)
16 W white LED spot light	16 W white LED spot light	16 W white LED spot light

Due to its industrial importance [7,73], the Schenk ene-reaction of citonellol (**49**) was studied for two concentrations of **49** of 0.1 M and 0.35 M. After reduction of the initially formed hydroperoxides with NaBH_4_ to the corresponding allyl alcohols **50** and **51**, yields and compositions were determined by ^1^H-NMR analysis *vs.* an internal standard. In all cases, **50** and **51** were obtained in a ratio of 1:1.5. The dual-channel reactor gave almost quantitative yields of 97% for both starting concentrations after just 3 min of irradiation. Gas and liquid flow could be furthermore controlled separately. Because of its lower contact area-to-volume ratio, the mono-channel system required longer residence times of 15 min and 31 min to reach high yields of 95% and 94%, respectively. The gas-liquid flow also proved to be difficult to control. In contrast to the microflow setups, it took 3 h and 8.5 h to achieve yields of 92% and 89% in the batch system. Daily output calculations were furthermore conducted for all three setups showing that the dual-channel reactor could generate more than 5-times (0.1 M of **49**) and over 10-times (0.35 M of **49**) the amount produced by the mono-channel. A scale-up was examined with a larger dual-channel reactor. Using a 0.35 M solution of **49**, a yield of 95% was achieved after a residence time of only 3 min. The daily output was calculated to 45.49 mmol d^−1^, even exceeding the values of the batch system (14.7 mmol d^−1^ and 17.6 mmol d^−1^).

The [4+2]-cycloaddition of α-terpinene (**45**) to ascaridole (**46**) was additionally carried out in the dual-channel reactor to investigate the effect of various reaction parameters. Using an optimized flow rate of O_2_ of 105 μL min^−1^, a starting concentration of **45** of 0.35 M and a residence time of 3 min, a yield of ascaridole (**46**) of 91% was achieved. The analogue batch reaction showed a reduced yield of 82% despite a prolonged irradiation period of 3 h.

The ene-reactions of the allylic alcohols **52** to the allylic hydroperoxides **53** were furthermore chosen to investigate the durability of the dual-channel reactor over an extended time period. After 4 days of continuous running, the reactor showed no drop in reaction efficiency and no operation problems. Using 1.17 M solutions of **52**, near complete conversions of ≥97% were accomplished after a residence time of 122 s. The hydroperoxide **53b** was obtained in a diastereoisomeric mixture of 75:25 (syn/anti).

#### 3.2.2. Photochlorinations of Cycloalkanes

**Scheme 16:**
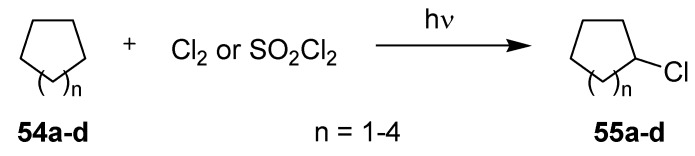
Photochlorination of cycloalkanes.

Recently, Ryu *et al.* have studied the photochlorination of cycloalkanes **54a–d** in various microflow setups (Scheme 16; Table 17) [74]. Monochlorination of cyclohexane (**54b**) to chlorocyclohexane (**55b**) was initially achieved in a stainless steel reactor with a Pyrex cover using ambient light and molecular chlorine. The reagent gas was introduced via a separate syringe pump and mixed with the cyclohexane stream in a T-shaped micromixer. The reaction mixture was subsequently pumped through the microflow reactor and the product was collected in a flask containing a 10% solution of sodium sulfite. After a residence time of 19 min, high selectivity for monochlorination (>95%) was achieved and chlorocyclohexane (**55b**) was obtained in a yield of 20% (by GC).

Likewise, the photoinduced chlorinations of various cycloalkanes **54a–d** with sulfuryl chloride as a less reactive chlorination agent were successfully realized using a 15 W black light source in combination with a glass based reactor. When a cycloalkane/SO_2_Cl_2_ solution of 40:1 was introduced and irradiated for 57 min, the corresponding chlorocycloalkanes **55a–d** were isolated in moderate to high yields of 35–87%. As would be expected, a reduction of residence time caused a drop in product yields. The reaction involving cyclohexane was furthermore performed with 2 × 15 W lamps, which gave an improved yield of **55b** of 45%.

**Table 17:** Key parameters of microreactors used. No batch reactions were performed.

Steel based μ-reactor	Glass based μ-reactor 2
Dainippon Screen (stainless steel, Pyrex window) 1,000 μm × 300 μm × 2.35 m (W × D × L) 0.7 mL (V_channel_)	mikroglas chemtech dwell device (Foturan™) 1,000 μm × 500 μm × 1.9 m (W × D × L) 0.95 mL (V_channel_)
ambient light	15 W black light

### 3.3. Scale-Up and Industrial Applications

The synthetic transformations described above have been mainly performed on micro-scales, demonstrating the feasibility of microflow photochemistry as a novel R&D tool. Simple strategies for scale-up include, among others, ‘numbering up’ or ‘clustering’ of various microreactors in parallel or in series mode, introduction of larger and wider reaction channels, continuous operation for long periods of time, introduction of larger volumes of reaction solutions and higher concentrations of reagents. Adopting these approaches, small laboratory scales have been realized, for example, for the [2+2]-cycloaddition of coumarin derivative **8** using the LOPHTOR system [53] or for the photo-oxygenation of citronellol (**49**) utilizing the dual-channel reactor [72]. The photodimerization of maleic anhydride (**43**) in the microcapillary-ultrasound setup was additionally achieved using a large stock solution of 300 g [68]. While current microflow photochemical technology may not be suitable for large industrial-scale productions (>1 t a^−1^), examples of low-volume processes, including an industrial one, have already been realized.

#### 3.3.1. Barton Reaction

Ryu and coworkers achieved a multigram synthesis of the steroidal oxime **7** using a DMF solution of **6** (Scheme 2; Table 18) and two stainless steel reactors in series coupled with 8 × 20 W black light lamps [52]. After continuous operation for 20 h with a residence time of 32 min, the isolated amount of product **7** was 3.1 g, equal to a yield of 60%. In an extension of this work and in collaboration with Dainippon Screen Mfg, the authors developed a prototype of a fully automated photo-microreactor system (DS-AMS-1) [52]. This reactor system offered easy operation and monitoring via PC-software together with other important safety features. The setup incorporated a single multi-lane stainless steel reactor with a Pyrex top. Irradiation was achieved with six evenly positioned 15 W black light sources. After constant operation for 40 h with a residence time of 20 min, the desired oxime **7** was obtained in an amount of 5.3 g, corresponding to an isolated yield of 61%.

**Table 18:** Key parameters of the advanced microreactor systems developed.

Multi-lane μ-reactor	Automated Multi-lane μ-reactor
Dainippon Screen (stainless steel, Pyrex window) 1,000 μm × 500 μm × 0.5 m (W × D × L) 16 lanes, 2 reactors in series8 mL (V_channel_)	Dainippon Screen DS-AMS-1 (stainless steel, Pyrex window) 1,000 μm × 500 μm × 0.5 m (W × D × L) 16 lanes 4 mL (V_channel_)
8 × 20 W black light	6 × 15 W black light

#### 3.3.2. Paternò-Büchi Reaction

**Scheme 17:**
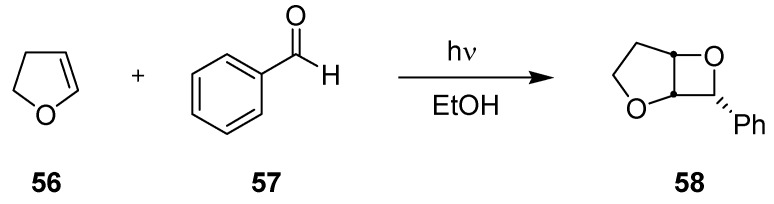
Paternò-Büchi reaction involving 2,3-dihydrofuran.

**Figure 4:**
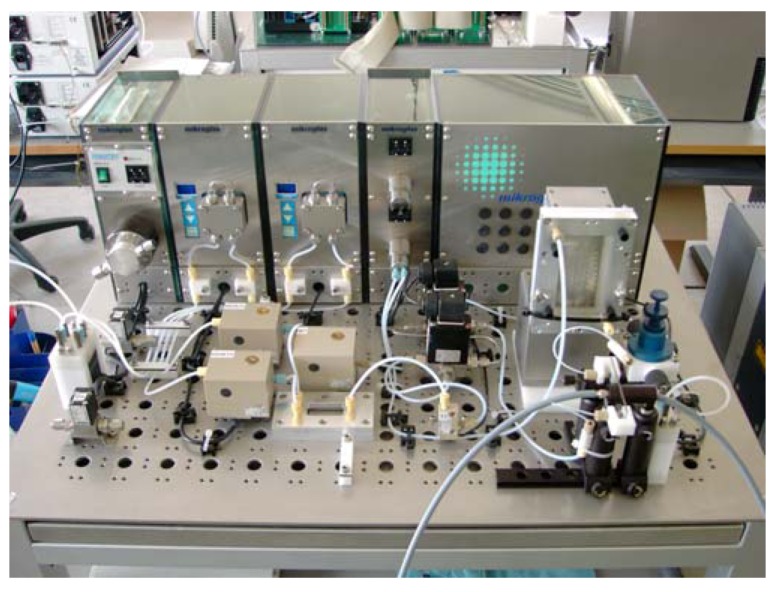
Automated microphotoreaction system (courtesy of mikroglas chemtech).

The [2+2]-photocycloaddition between 2,3-dihydrofuran (**56**) and benzaldehyde (**57**) in an automated and computer controlled microreaction plant has been briefly described by Freitag *et al.* (Scheme 17; Figure 4) [75]. The system had two inlet channels for reagents, one for inert gas and one for solvent (used for ‘rinsing’). The reagents and the inert gas were mixed in a premixer and pumped into the reaction loop. This loop incorporated a dwell device attached to a compact 308 nm excimer cube. An integrated IR-sensor with a flow cell attached to an external FT-IR spectrometer continuously recorded the progress of the reaction. At maximum conversion, an automated valve opened and the product mixture was eluted from the loop and collected at the outlet. On demand, temperature control was achieved with an external cooling circuit. Using this device, a daily output of approximately 1 kg d^−1^ of the oxetane **58** has been estimated.

#### 3.3.3. Photoisomerization of Camptothecin-*N*-oxides

**Scheme 18:**
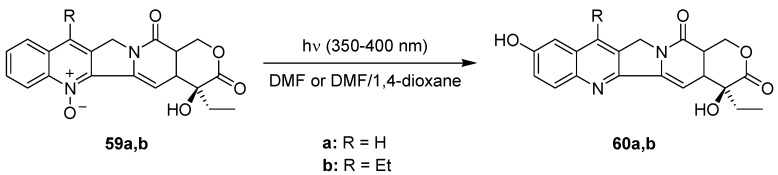
Synthesis of camptothecin derivatives.

The first industrial photochemical process involving a multi microphotoreactor setup was realized by Heraeus Noblelight [76]. The plant is currently used for the kg-scale synthesis of 10-hydroxycamptothecin (**60a**) and 7-ethyl-10-hydroxycamptothecin (**60b**) from their corresponding *N*-oxides **59** (Scheme 18).

**Figure 5:**
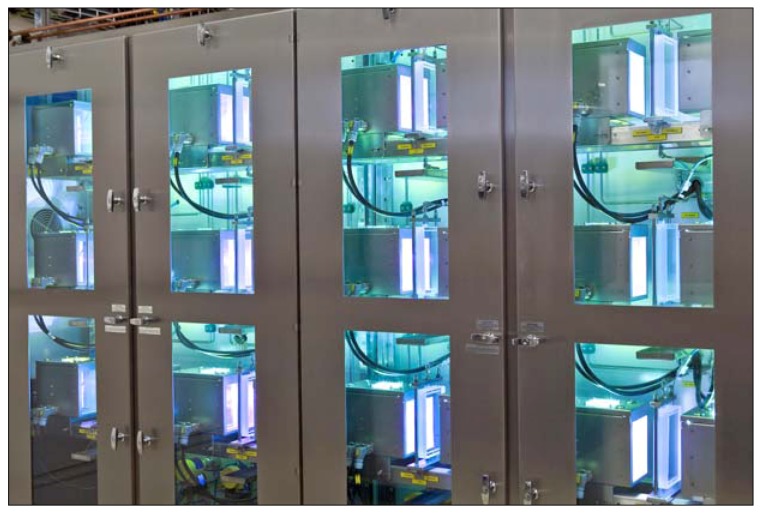
Photochemical production plant (courtesy of Heraeus Noblelight).

The product compounds **60** serve as precursors to the anti-cancer drugs irinotecan and topotecan with an annual demand below 1 t a^−1^. The photochemical production plant was able to meet this need as it allowed for a production of 2 kg of 10-hydroxycamptothecin (**60a**) per day, utilizing 330 kg of reaction solution. Under microflow conditions, the conversion of **59a** and yield of **60a** were high, 95% and 90%, respectively. A starting concentration of 0.6 weight-% of **59a** was furthermore feasible. In contrast, a conventional batch reactor gave a much lower conversion of **59a** and yield of **60a** of 85% and 50%, respectively. In addition, the batch mode required a 6-times higher dilution of 0.1 weight-% of **59a**. The developed plant consisted of twelve microreactor units operated in parallel and incorporating individual lamps (Figure 5) [76]. Each microreactor was made of two parallel quartz glass plates separated by a thin spacer. This design feature created a very thin film of the reaction solution (40–100 μm). Compact high pressure mercury lamps with spectral filters (350–400 nm) and an optical power output of 250 W were positioned on both sides of the microfilm reactor.

## 4. Conclusions

Microflow photochemistry efficiently combines the advantages of microspace and flow conditions. Consequently, a broad range of photochemical transformations has been successfully realized. Microchemical processes commonly result in the reduction of irradiation times, enhanced selectivity and increased light efficiency, thus unambiguously demonstrating the superiority of microflow photochemistry over conventional techniques. It is envisaged that this new synthesis concept will emerge as an important future R&D and production methodology. The industrial Heraeus Noblelight process evidently demonstrates this potential. Since the submission of this article, additional examples of photochemical reactions in microflow devices have been published, thus demonstrating the growing importance of microflow photochemistry [77,78,79,80,81]. Ultimately, microflow photochemistry may emerge as ‘the New Photochemistry of the Future’ [82].
